# Association Between Arterial Hypertension and Periodontitis: A Systematic Review and Meta-Analysis

**DOI:** 10.3390/jcm15155930

**Published:** 2026-07-29

**Authors:** Ani Matinyan-Hakobyan, Beatriz Gonzalez-Navarro, Sonia Egido-Moreno, Fabiola Mazza-Padrón, Anna Oliveras-Serrano, Jose López-López

**Affiliations:** 1 Department of Stomatology, Master of Medicine, Surgery and Oral Implantology, Faculty of Medicine and Health Sciences, UB-Odontology Hospital University of Barcelona, C/Feixa Llarga s/n, 08907 L’Hospitalet de Llobregat, Spain; amatinma8@alumnes.ub.edu (A.M.-H.); soniaegido@ub.edu (S.E.-M.); jl.lopez@ub.edu (J.L.-L.); 2 Bellvitge Biomedical Research Institute (IDIBELL), 08908 L’Hospitalet de Llobregat, Spain; 3 Barcelona Dental Hospital [HOUB], 08970 Barcelona, Spain; 4 Hypertension and Vascular Risk Unit, Department of Nephrology, Hospital del Mar, University Pompeu Fabra, 08003 Barcelona, Spain; aoliveras@hmar.cat

**Keywords:** hypertension, periodontitis, oral health, cardiovascular risk

## Abstract

**Background**: Periodontitis and arterial hypertension are highly prevalent chronic conditions that share common risk factors and inflammatory pathways. Increasing evidence suggests a potential association between both diseases; however, the strength and consistency of this relationship remain unclear. **Methods**: A systematic review and meta-analysis of observational studies was conducted following PRISMA guidelines. Electronic searches were performed in PubMed (MEDLINE), Scopus, and the Cochrane Library without date restrictions. Studies evaluating the association between periodontitis and arterial hypertension in humans were included. Risk of bias was assessed using the Newcastle–Ottawa Scale. A random-effects meta-analysis was performed using adjusted odds ratios (ORs). **Results**: Thirty-six studies were included in the systematic review; of these, 11 studies reporting a directly comparable, adjusted odds ratio were included in the quantitative meta-analysis, comprising a total of 48,761 participants. The pooled analysis demonstrated a statistically significant association between periodontitis and hypertension (OR = 1.65; 95% CI: 1.42–1.93; *p* < 0.00001). Heterogeneity was substantial (I^2^ = 65%) but was partly explained by periodontal case definition, with a stronger association among studies using the AAP/EFP classification (OR = 1.90; 95% CI: 1.57–2.30) than those using alternative periodontal indices (OR = 1.35; 95% CI: 1.21–1.50). The overall certainty of evidence, assessed using GRADE, was rated as Very Low. **Conclusions**: This systematic review and meta-analysis identifies a consistent positive association between periodontitis and arterial hypertension across observational studies. Given the very low certainty of the underlying evidence, this finding should be interpreted as a signal warranting further investigation rather than a robust, settled conclusion. Causality cannot be established from the available observational evidence, and the potential relevance of periodontal health to cardiovascular risk merits further research.

## 1. Introduction

Arterial hypertension is a major global public health concern and one of the leading modifiable risk factors for cardiovascular morbidity and mortality. It is defined as a sustained elevation of blood pressure, typically ≥140/90 mmHg, and is associated with an increased risk of coronary artery disease, stroke, heart failure, and chronic kidney disease [1]. Its pathogenesis is multifactorial, involving the interaction of renal, neural, vascular, endocrine, genetic, and environmental factors [2]. Chronic inflammation and endothelial dysfunction are recognized as central mechanisms contributing to vascular remodeling and increased peripheral resistance.

Periodontitis is a chronic multifactorial inflammatory disease caused by a dysbiotic subgingival biofilm that leads to destruction of the tooth-supporting structures, resulting in clinical attachment loss and alveolar bone resorption [3]. Beyond its local effects, periodontitis has been associated with increased systemic inflammatory burden, including elevated circulating levels of markers such as C-reactive protein (CRP) and interleukin-6. Periodontitis disproportionately affects individuals of lower socioeconomic status and has a significant negative impact on quality of life [4].

Hypertension and periodontitis are among the most prevalent chronic diseases worldwide, each affecting approximately one in three adults, and their prevalence increasing with age [5]. According to the World Health Organization (WHO), hypertension contributes to about 51% of deaths from stroke and 45% of deaths from cardiovascular disease globally [6]. Periodontitis, meanwhile, is a major cause of tooth loss in adults and affects around 30–50% of the global population, with severe forms present in about 10% [7]. Importantly, both conditions share several modifiable risk factors, including smoking, diabetes mellitus, obesity, psychosocial stress, and unhealthy lifestyle behaviors [8].

The high prevalence and shared risk factors of hypertension and periodontitis have attracted increasing scientific interest. Both conditions are associated with systemic inflammation, oxidative stress, endothelial dysfunction, and dysregulation of immune and vascular pathways [9]. Epidemiological studies suggest that individuals with moderate or severe periodontitis tend to have higher blood pressure and are 30–70% more likely to develop hypertension than periodontally healthy individuals [10]. Emerging evidence also indicates that periodontal therapy may reduce systolic and diastolic blood pressure.

In recent years, the relationship between periodontitis and cardiovascular diseases, including hypertension, has been intensively studied. Prior literature has proposed a potential bidirectional association: hypertension has been suggested as a possible risk factor for periodontitis, while chronic periodontal inflammation may contribute to increased blood pressure through systemic inflammatory and immunological pathways [11,12,13]. However, these two directions, along with related but distinct outcomes such as blood-pressure control status, have often been analyzed together in prior reviews without clearly distinguishing them. Experimental and interventional studies further suggest that treating periodontitis may improve vascular endothelial function and lower blood pressure [14], although the precise mechanisms linking periodontal infection to hypertension remain to be fully elucidated.

Given the global burden of both conditions and their potential interrelationship, oral health professionals can play a pivotal role in the early detection and prevention of hypertension. Increasing awareness of this association could promote integrated, multidisciplinary strategies for cardiovascular risk reduction [15].

The purpose of this systematic review and meta-analysis is to evaluate the association between periodontitis and hypertension in adult populations, synthesizing the most relevant and recent evidence. The secondary objectives are to highlight the importance of early diagnosis and comprehensive management of both diseases and to identify shared risk factors and potential mechanisms linking them.

## 2. Materials and Methods

The present systematic review and meta-analysis adheres to “Preferred Reporting Items for Systematic Reviews and Meta-Analyses” (PRISMA) guidelines [16] (Supplementary Materials). Table 1 shows the individual parts of the PECOS question.

The protocol of this systematic review was registered in the PROSPERO database (International Prospective Register of Systematic Reviews) under the registration number CRD420261355740 in April 2026, prior to data extraction and analysis.

### 2.1. Focused Question and PECOS Items

The focused question to be addressed was: Is there an association between periodontitis and arterial hypertension in adult populations? Accordingly, our PECOS (Patients, Exposure, Comparison, Outcomes, Studies designs) items were:

- (P) Patient: Adults (≥18 years), with or without hypertension.
- (E) Exposure: Presence of periodontitis (diagnosed clinically by probing depth, clinical attachment loss, bleeding on probing, or validated periodontal case definitions).
- (C) Comparison: Individuals without periodontitis (periodontally healthy or without defined periodontal disease).
- (O) Outcomes: Arterial hypertension (defined by systolic/diastolic blood pressure thresholds, medical diagnosis, or antihypertensive medication use).
- (S) Study design: Observational studies, including cross-sectional, case–control, and cohort designs.

### 2.2. Eligibility Criteria

Studies were included if they met the following criteria: observational studies, including cross-sectional, case–control, or cohort designs; studies conducted in human adult populations (≥18 years); studies evaluating the association between periodontitis and arterial hypertension and/or blood pressure levels; studies with a clinical diagnosis of periodontitis based on established periodontal parameters, such as probing depth, clinical attachment loss, bleeding on probing, or validated case definitions; and studies reporting hypertension based on clinical diagnosis, blood pressure measurements, or the use of antihypertensive medication.

Studies using tooth loss, edentulism, or oral-hygiene indices as a proxy for periodontal disease were also eligible for inclusion in the systematic review and narrative synthesis, given their relevance to the broader oral health–hypertension literature, but were not eligible for the quantitative meta-analysis.

### 2.3. Search Strategy

Two independent reviewers (A.M.-H. and B.G.-N.) conducted a comprehensive literature search in PubMed (MEDLINE), Scopus, and Cochrane Library databases, initially in September 2025 and updated in March 2026 prior to resubmission, with no restriction on publication date or language, using the following search strategy:

- PubMed: (“hypertension”[MeSH Terms] OR “hypertension”[All Fields] OR (“high”[All Fields] AND “blood”[All Fields] AND “pressure”[All Fields]) OR “high blood pressure”[All Fields] OR “hypertense”[All Fields] OR “hypertension s”[All Fields] OR “hypertensions”[All Fields] OR “hypertensive”[All Fields] OR “hypertensive s”[All Fields] OR “hypertensives”[All Fields]) AND (“periodontal diseases”[MeSH Terms] OR “periodontitis”[MeSH Terms] OR “periodontal”[All Fields] OR “periodontally”[All Fields] OR “periodontically”[All Fields] OR “periodontics”[MeSH Terms] OR “periodontics”[All Fields] OR “periodontic”[All Fields] OR “periodontitis”[All Fields] OR “periodontitides”[All Fields] OR “periodontal disease”[All Fields] OR “periodontal diseases”[All Fields])
- Scopus: TITLE-ABS-KEY ((“Hypertension” OR “Blood Pressure” OR hypertension OR “high blood pressure”) AND (“Periodontal Diseases” OR periodontitis OR “periodontal disease”)) AND (LIMIT-TO (DOCTYPE, “ar”))
- Cochrane Library: High blood pressure OR hypertension (explode all trees) AND periodontitis (explode all trees)

### 2.4. Selection of Studies

Two independent reviewers (A.M.-H. and B.G.-N.) selected the studies in accordance with the previously established inclusion criteria. We followed the screening steps described in the PRISMA statement [16]. The reviewers first screened all records based on titles and abstracts, and subsequently assessed the full texts of potentially eligible studies against the inclusion criteria. Disagreements between the two reviewers at any stage of selection were resolved by consensus discussion; no third reviewer was required. A PRISMA flow diagram was made to summarize the selection process.

### 2.5. Data Extraction

Data extraction was completed independently by both authors (A.M.-H. and B.G.-N.), with discrepancies resolved by consensus discussion. For the selected studies, the two authors recorded whenever possible: author(s), year of publication, study design, country, details of participants (total of patients and mean age), periodontitis and hypertension definitions and assessment methods. A second table was made capturing, for each study, the effect estimate type and value with its 95% confidence interval (or *p*-value when a confidence interval was not reported), covariate adjustment, whether the study was included in the primary quantitative meta-analysis, and, when not pooled, the specific reason (e.g., incompatible effect measure, non-validated periodontal exposure, reversed exposure-outcome direction, or different outcome construct). When a study reported multiple adjustment models, the most fully adjusted model was extracted preferentially.

### 2.6. Risk of Bias Assessment

As part of the data extraction process, two reviewers (A.M.-H. and B.G.-N.) independently assessed the risk of bias of all included studies. Given that most included studies were cross-sectional rather than cohort or case–control in design, the original Newcastle–Ottawa Scale (NOS) [17] was applied inconsistently across designs in our initial assessment. We therefore applied three design-specific instruments:

- The standard NOS for cohort studies, applied to the studies whose reported association reflects a genuine longitudinal, incident-outcome analysis. Items evaluated: (1) representativeness of the exposed cohort, (2) selection of the non-exposed cohort, (3) ascertainment of exposure, (4) demonstration that the outcome was not present at baseline, (5–6) comparability of cohorts, (7) assessment of outcome, (8) adequacy of follow-up duration, (9) adequacy of follow-up completeness.
- The Herzog et al. (2013) [18] adaptation of the NOS for cross-sectional studies, applied to the studies whose reported association reflects a single-timepoint (prevalence-based) analysis. Domains: Selection (representativeness of the sample, sample-size justification, ascertainment of non-respondents, ascertainment of exposure; maximum 5 stars), Comparability (maximum 2 stars), Outcome (assessment of outcome, statistical test; maximum 3 stars).
- The standard NOS for case–control studies, evaluated: (1) case definition adequacy, (2) representativeness of cases, (3) selection of controls, (4) definition of controls, (5–6) comparability, (7–8) ascertainment of exposure, (9) non-response rate.

Risk of bias was categorized as low (7–9 stars), moderate (4–6 stars), or high (0–3 stars) for cohort and case–control studies, using the standard Newcastle–Ottawa Scale cutoffs. For cross-sectional studies, assessed using the Herzog-modified NOS (maximum 10 stars), studies were categorized as low (8–10 stars), moderate (6–7 stars), or high (≤5 stars) risk of bias, as no universally accepted cutoff exists for this adapted instrument.

### 2.7. Statistical Analysis

The primary meta-analysis was restricted to studies reporting a genuine adjusted odds ratio (OR) for periodontitis (exposure) and prevalent or incident hypertension (outcome), consistent with the primary relationship defined in our eligibility criteria. Studies reporting prevalence ratios, risk ratios, incidence-rate ratios, hazard ratios, or beta-coefficients for continuous blood pressure were not statistically interchangeable with an OR, particularly given that hypertension was common rather than rare in most included populations, and were therefore excluded from quantitative pooling; these studies, together with those assessing a reverse-direction association, a different outcome construct, or a non-validated periodontal exposure (e.g., tooth loss, edentulism, or oral-hygiene indices), are reported narratively.

Data analysis was performed using RevMan Web (Review Manager Web, The Cochrane Collaboration: https://revman.cochrane.org, accessed on 25 June 2026). Combined effect measures were calculated using a random-effects model (inverse-variance method) with restricted maximum likelihood (REML) estimation of τ^2^, applied to log-transformed adjusted odds ratios, given the anticipated heterogeneity across studies.

Heterogeneity was evaluated using Cochran’s Q, its associated *p*-value, I^2^, and τ^2^, reported as distinct statistics. An I^2^ value > 50% was interpreted as substantial heterogeneity.

Subgroup analyses were performed by periodontal case definition and hypertension ascertainment method. Subgroup analysis by study design was not performed for the primary pool, as only one included study using a genuine odds ratio employed a cohort design; the cohort studies using hazard ratios or risk ratios are instead discussed narratively. A formal meta-regression was not performed given the limited number of pooled studies [10], which provides insufficient power for reliable covariate estimation.

Egger’s test, Begg’s rank-correlation test, leave-one-out sensitivity analysis, and the 95% prediction interval were calculated using R version 4.6.1 (R Core Team) with the meta package.

### 2.8. Certainty of Evidence Assessment (GRADE)

The certainty of evidence for the primary meta-analyzed outcome was assessed using the GRADE (Grading of Recommendations Assessment, Development and Evaluation) framework [19]. Two reviewers independently rated the certainty of evidence across the five standard GRADE domains: risk of bias, inconsistency, indirectness, imprecision, and publication bias. Disagreements were resolved by discussion or consultation with a third reviewer.

The certainty of evidence started at Low, as it is derived exclusively from observational studies, and could be downgraded by one or two levels per domain if serious or very serious concerns were identified (e.g., substantial heterogeneity for inconsistency, wide confidence intervals or a prediction interval crossing the null for imprecision, or asymmetry on Egger’s test for publication bias), or upgraded in the presence of a large magnitude of effect, a dose–response gradient, or plausible confounding that would reduce the observed association.

## 3. Results

### 3.1. Study Selection

**Figure 1:**
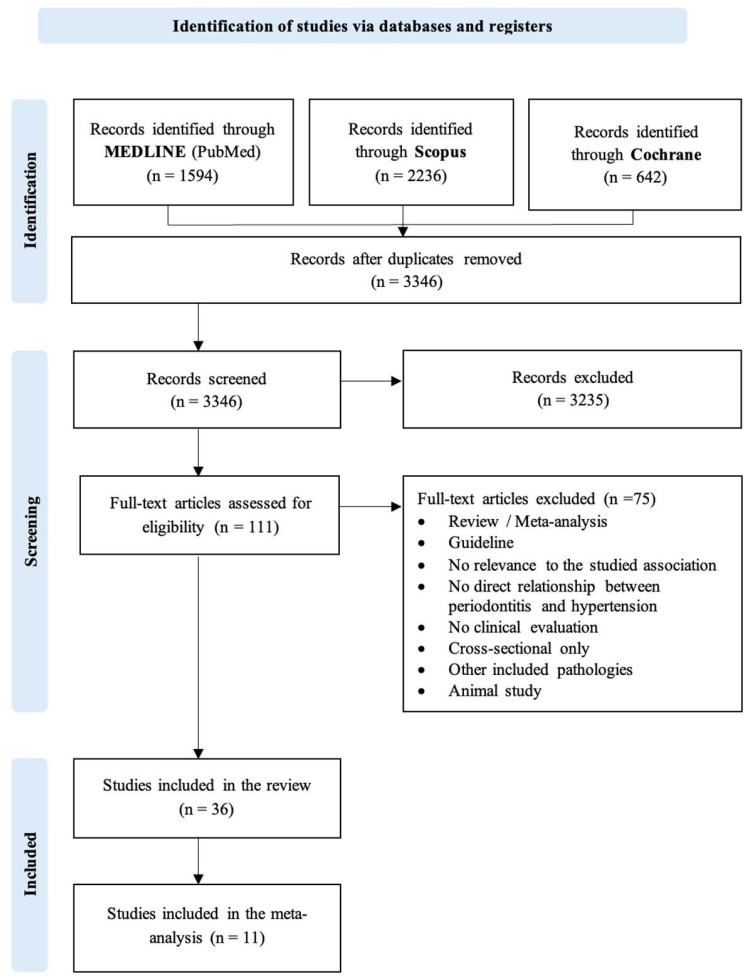
PRISMA flow diagram.

Figure 1 shows the initial electronic database research PRISMA flow diagram that put 4472 articles on display. Out of 4472 articles identified in the electronic search, 1126 were identified as duplicates and were removed, leaving 3346 articles for record screening.

After reading the abstracts and considering the inclusion and exclusion criteria we selected 111 of them to be evaluated completely. After full text reading, 15 studies were excluded due to being literature reviews, 14 for not showing clinical evaluation, 6 for being animal studies, 7 for being guidelines, 3 for not having relevance to the studied association, 7 for not showing sufficient data, 9 for not having a direct relationship between periodontitis and hypertension, and 14 for including other pathologies. Finally, a total of 36 studies met the eligibility criteria and were included in the systematic review and narrative synthesis [20,21,22,23,24,25,26,27,28,29,30,31,32,33,34,35,36,37,38,39,40,41,42,43,44,45,46,47,48,49,50,51,52,53,54,55]; of these, 11 studies reporting a comparable, adjusted odds ratio for the same exposure-outcome relationship were included in the quantitative meta-analysis. The level of agreement between reviewers was good with a kappa index of 0.7937.

### 3.2. Study Characteristics

A total of 36 studies published between 2006 and 2025 were included as shown in Table 2. Study designs were predominantly cross-sectional designs (*n* = 28) [20,21,22,23,25,26,27,29,30,31,32,33,34,35,37,38,39,40,41,43,45,46,48,49,50,53,54,55], a few prospective cohort studies (*n* = 6) [28,36,42,44,47,52], one nested case–control study [24], and one single-arm interventional (pre–post) study [51]. The majority of studies were conducted in Asia, followed by Europe, North America and Africa, reflecting a broad geographic representation with a total of 20 different countries. Several cross-sectional studies employed large population-based samples derived from national surveys or administrative databases [30,42,54], whereas cohort studies generally involved smaller but well-characterized populations.

Sample sizes varied widely, ranging from 26 [51] to 709,584 participants [42] (average of 26,829), totaling 982,300 subjects across the 36 studies (mean per-study sample size: 27,286). A precise overall mean age could not be calculated for the full sample, as several studies reported only an age range or did not specify age. Among the 24 studies reporting a mean age, values ranged from 31.0 years [53] to 76.0 years [44], with an unweighted mean of 51.7 years. Among the 23 studies reporting a sex breakdown, the overall sample was balanced, with women representing approximately 49.5% of participants. Participants were recruited from community-based settings, healthcare facilities, or national health surveys, depending on study design.

Considerable heterogeneity was observed in the definition of periodontitis. Diagnostic criteria included the 2017 World Workshop AAP/EFP classification [21,24,26,53,54]; the 2018 World Workshop AAP/EFP classification [23,38,43,45]; the Community Periodontal Index/Treatment Needs (CPI/CPITN), applied either as a categorical index [22,25,29,30,35,41,42,47,55], with a CPI ≥ 4 cutoff [31,32,36,48,49], or without a stated cutoff [40]; CDC/AAP (2012) case definitions [28,44,51]; the Swedish National Dental Insurance Index (SNDI) [33]; the criteria of Eke and Page [37]; Russell’s Periodontal Index [50]; and tooth-loss-based definitions, e.g., ≥10 missing teeth [20,27,34,39,46]. Periodontal status ascertainment was not specified in one study [52]. Despite these methodological differences, all included studies provided a categorical comparison between participants with and without periodontitis (or, where applicable, with and without significant tooth loss).

Hypertension was most defined using an SBP and/or DBP threshold (≥140/90 mmHg or >140/90 mmHg), alone or combined with antihypertensive treatment or a self-reported/physician diagnosis (*n* = 32). Two studies defined hypertension solely on the basis of antihypertensive treatment [33,42,53,54]. In most studies, blood pressure was measured using standardized clinical methods, including mercury sphygmomanometers or validated automated devices (e.g., Omron M5-1), typically with two or three measurements per protocol. One study employed 24-h ambulatory blood pressure monitoring (ABPM) [51].

### 3.3. Data Extraction

Across the 36 included studies, the reported effect measures varied according to study design and analytical approach (Table 3). Most studies (*n* = 18) reported an odds ratio (OR) for a categorical comparison between participants with and without periodontitis; of these, 11 reported a directly poolable, adjusted OR for the same exposure-outcome relationship (periodontitis status vs. hypertension status) and were included in the quantitative meta-analysis. The remaining studies reported effect measures that could not be combined with the pooled OR without introducing bias: eight studies reported a β-coefficient for a continuous blood-pressure outcome rather than a categorical hypertension diagnosis; two studies derived a hazard ratio (HR) from Cox proportional-hazards regression for incident hypertension; two further studies reported a risk ratio (RR) from a comparable survival-analysis approach; one study reported an incidence rate ratio (IRR); one reported a prevalence ratio (PR); and three studies reported only a mean difference in blood pressure between exposure groups, without an OR.

Most analyses demonstrated statistically significant associations between periodontitis (or related oral health indicators) and blood pressure outcomes or hypertension status, with *p*-values commonly reported as <0.05 or <0.001. In several studies, statistical significance was not reported numerically; however, authors described a positive association between periodontitis-related measures and blood pressure outcomes after multivariable adjustment. This highlights heterogeneity in reporting practices rather than inconsistency in the direction of findings.

Beyond effect-measure incompatibility, several studies were excluded from pooling for construct-related reasons. In three studies, the exposure-outcome relationship was reversed relative to the review question, with hypertension status used as the grouping variable and a periodontal parameter analyzed as the outcome [25,45,50]. One study [51] was a single-arm interventional pre–post study evaluating the effect of periodontal treatment on blood pressure in already-hypertensive patients and was therefore not compatible with pooling alongside observational association studies. Another article [21], compared blood-pressure outcomes across periodontitis severity strata without an unaffected comparison group, and finally a study [23] assessed uncontrolled hypertension within an exclusively hypertensive subsample, a different outcome construct from hypertension status itself.

Tooth loss or edentulism was used as a proxy exposure for oral disease burden in five studies [20,27,34,39,46]. These were synthesized narratively rather than pooled, since tooth loss and clinically diagnosed periodontitis are related but non-identical exposure constructs. Findings across these studies were mixed: three reported a significant, positive association between tooth loss and higher blood pressure or hypertension prevalence [20,39,46], while two reported associations that were attenuated or lost statistical significance after multivariable adjustment [27,34].

Among the 11 studies included in the quantitative pool, all reported a positive point estimate for the periodontitis–hypertension association, and 10 of 11 reached statistical significance at the study level (95% CI); the exception [32], showed a positive but non-significant association (OR 1.95, 95% CI 0.89–4.91), consistent with its comparatively small sample size (*n* = 420). Among studies not eligible for pooling, the overall direction of association was similarly, though not universally, positive: most β-coefficient and alternative-effect-measure studies reported a positive association with continuous blood pressure or incident hypertension, while two Cox-regression-derived estimates were null for periodontitis specifically [44,52].

Across the 36 studies, adjustment for age and sex was near-universal, and most models additionally adjusted for smoking status and body mass index. A subset of studies further incorporated socioeconomic or lifestyle variables (e.g., income, education, alcohol consumption) or inflammatory biomarkers such as C-reactive protein, reflecting growing attention to shared inflammatory pathways as a candidate mechanism linking periodontitis and hypertension.

Longitudinal evidence, although not eligible for pooling due to effect-measure incompatibility, offered mixed support for temporality. Two Cox-regression analyses reported an increased hazard of incident hypertension among individuals with periodontitis or related exposures at baseline [42,47], while two others reported null associations for periodontitis specifically after adjustment [44,52], indicating that longitudinal evidence for this relationship remains mixed rather than uniformly confirmatory.

### 3.4. Risk of Bias Assessment

Overall, the risk of bias across the included studies was predominantly low-to-moderate, with high-risk ratings mainly driven by limited representativeness, reduced control for confounding, and/or incomplete reporting of key methodological aspects. Studies classified as low risk of bias typically demonstrated adequate exposure and outcome assessment and appropriate adjustment for confounders. The methodological quality of the 36 observational studies included in this systematic review was evaluated using three design-specific adaptations of the Newcastle–Ottawa Scale (NOS) [17]: the standard NOS for cohort studies (maximum 9 points) for the six studies with a prospective cohort design [28,36,42,44,47,52]; the adaptation of the NOS for cross-sectional studies (maximum 10 points) for the 28 cross-sectional studies; and the standard NOS for case–control studies (maximum 9 points) for the single nested case–control study [24]. The single interventional study [51] was not eligible for NOS assessment and was appraised narratively. Because the three instruments differ in structure and maximum achievable score, ratings were interpreted relative to each design’s own scale rather than by direct numerical comparison across designs. Overall, 23 of the 35 rated studies were classified as low risk of bias, 11 as moderate risk, and 1 as high risk (Table 4, Table 5 and Table 6).

In the Selection domain, representativeness was generally stronger among studies drawing on national health surveys or large population-based cohorts [25,37,40,43,48,54]. Conversely, hospital-based or small convenience samples showed clearer limitations in external validity, most notably [26,41,45,50], all of which scored poorly on representativeness, sample-size justification, and description of non-respondents. Exposure ascertainment was adequate in most studies, which relied on a full-mouth clinical periodontal examination; ascertainment was comparatively weaker in the five studies using tooth loss as a proxy exposure rather than a direct periodontal examination [20,27,34,39,46].

The Comparability domain was consistently one of the strongest components across the evidence base. Nearly all studies adjusted for age and sex as a minimum, and most additionally accounted for at least one cardiometabolic or lifestyle covariate, such as smoking status, body mass index, diabetes, alcohol consumption, or socioeconomic indicators. There was one single exception [26], which reported only a descriptive comparison without multivariable adjustment. Nevertheless, residual confounding cannot be excluded given the heterogeneity in the selection and measurement of covariates across studies.

In the Outcome domain, ascertainment of blood pressure and hypertension status was generally robust, with most studies relying on standardized clinical measurement protocols and internationally recognized diagnostic thresholds. The six cohort studies achieved the highest ratings on outcome-related items, reflecting their ability to demonstrate that the outcome was absent at baseline and to assess incident hypertension over a defined follow-up period. Follow-up-related items were, by definition, not applicable to the 28 cross-sectional studies, reflecting an inherent design limitation in establishing temporality between periodontitis and hypertension.

Overall, risk of bias across the included studies was predominantly low, and no study was excluded from the systematic review on quality grounds alone. The single high-risk study [41], was primarily limited by poor representativeness of its small, hospital-based sample and did not report an odds ratio for the periodontitis–hypertension association. Studies rated as low risk of bias consistently combined adequate exposure and outcome ascertainment with appropriate adjustment for confounders, supporting the overall reliability of the evidence synthesized before.

### 3.5. Meta-Analysis of Included Studies

**Figure 2:**
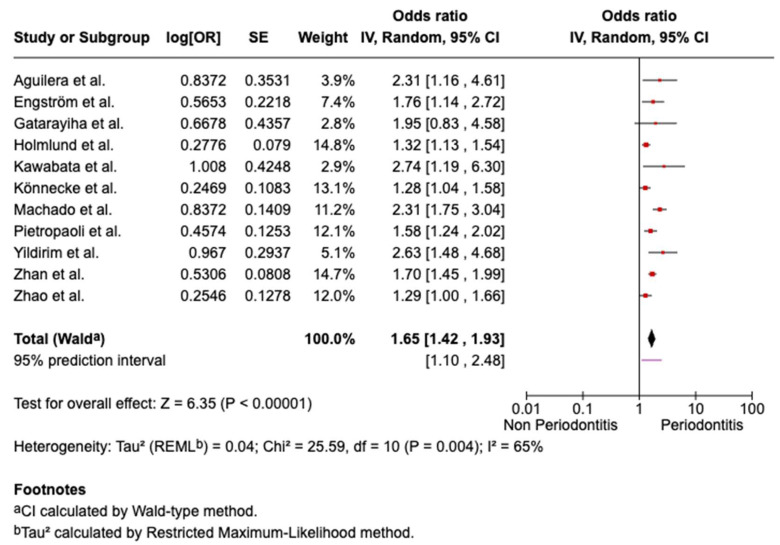
Forest plot of the association between periodontitis and hypertension across the 11 pooled studies (random-effects model, inverse-variance weighting, REML). OR > 1 indicates higher odds of hypertension among individuals with periodontitis. Each dot represents an individual study; the solid vertical line indicates no effect (OR = 1); the diamond represents the pooled effect estimate.

Eleven studies reporting a directly comparable, adjusted odds ratio (OR) for the association between periodontitis and hypertension were pooled using a random-effects model (REML, RevMan Web) [24,31,32,33,36,37,38,43,53,54,55]. The pooled analysis showed a significant positive association between periodontitis and hypertension (OR 1.65, 95% CI 1.42–1.93, Z = 6.35, *p* < 0.00001) (Figure 2). Individual study weights in the random-effects model ranged from 2.8% [32] to 14.8% [33], with no single study contributing a disproportionate share of the pooled estimate.

Heterogeneity among the pooled studies was substantial (I^2^ = 65%, Q = 25.59, df = 10, *p* = 0.004, τ^2^ = 0.04), consistent with the clinical and methodological diversity of the included populations, periodontal case definitions, and hypertension ascertainment methods described before. Leave-one-out sensitivity analysis confirmed that the pooled estimate was not driven by any single study: omitting individual studies produced pooled ORs ranging from 1.55 to 1.71, all remaining statistically significant, with I^2^ ranging from 46.3% to 64.8%. The 95% prediction interval, reflecting the expected range of the true effect in a new, similar population, was 1.10–2.48 and did not cross the null, indicating that a positive association is expected in most comparable settings despite the observed heterogeneity.

**Figure 3:**
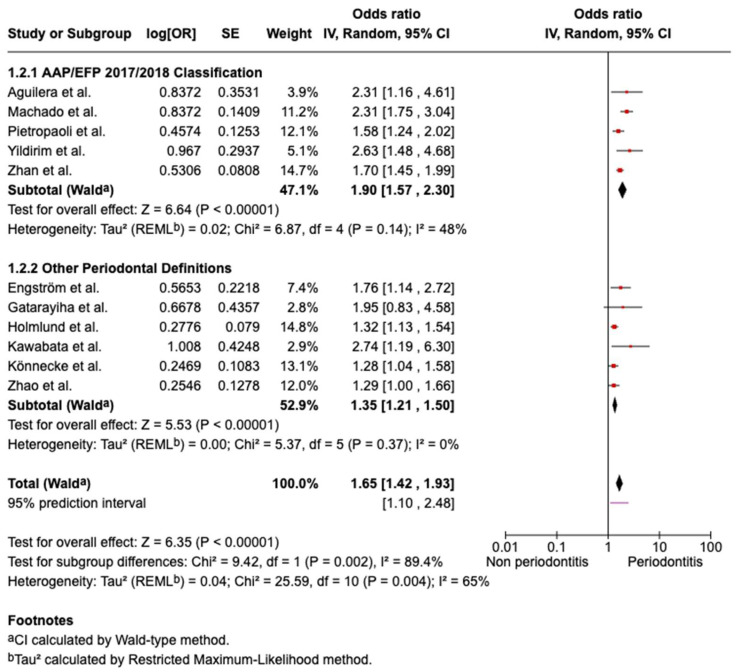
Subgroup forest plot by periodontal case definition (AAP/EFP classification vs. alternative indices), showing a statistically significant difference in effect size between subgroups. Each dot represents an individual study; the solid vertical line indicates the pooled effect estimate. The diamond represents the pooled effect estimate.

A pre-specified subgroup analysis by periodontal case definition partially explained the observed heterogeneity. Studies using the 2017/2018 World Workshop AAP/EFP classification [24,38,43,53,54] showed a stronger pooled association (OR 1.90, 95% CI 1.57–2.30, I^2^ = 48%) than studies using alternative periodontal indices or thresholds [31,32,33,36,37,55] (OR 1.35, 95% CI 1.21–1.50, I^2^ = 0%). This difference was statistically significant (χ^2^ = 9.42, df = 1, *p* = 0.002) (Figure 3). Notably, heterogeneity within each subgroup was markedly lower than in the overall pool, suggesting that the periodontal case definition used is a meaningful source of between-study variability, with the standardized AAP/EFP classification associated with a larger effect estimate. Given the limited number of studies per subgroup, a formal meta-regression was not performed, consistent with the a priori analytical plan.

Subgroup analysis by hypertension ascertainment method did not reveal a significant difference: studies using an objective SBP/DBP threshold, with or without an antihypertensive-medication criterion (*n* = 8), showed a pooled OR of 1.68 (95% CI 1.37–2.06, I^2^ = 60%), while studies relying on self-reported diagnosis or treatment status alone (*n* = 3) showed OR 1.65 (95% CI 1.22–2.25, I^2^ = 82%) (χ^2^ = 0.01, df = 1, *p* = 0.94).

A sensitivity analysis restricted to the nine studies rated low risk of bias yielded a pooled OR of 1.60 (95% CI 1.37–1.86, I^2^ = 66%), not significantly different from the two moderate-risk studies (χ^2^ = 2.50, df = 1, *p* = 0.11).

A subgroup analysis by sample size revealed a statistically significant small-study effect: studies with fewer than 3000 participants (*n* = 5) showed OR 2.17 (95% CI 1.77–2.67, I^2^ = 0%), compared with OR 1.47 (95% CI 1.29–1.68, I^2^ = 51%) among studies with 3000 or more participants (*n* = 6) (χ^2^ = 9.75, df = 1, *p* = 0.002), a pattern discussed in relation to publication bias below.

Geographic region was not associated with a significant difference: studies from Europe (*n* = 5, OR 1.64, I^2^ = 77%), Asia (*n* = 4, OR 1.76, I^2^ = 70%) and the two remaining studies from other continents (OR 1.61, 95% CI 1.27–2.03, I^2^ = 0%), showed overlapping estimates (χ^2^ = 0.22, df = 2, *p* = 0.90).

A subgroup analysis by design (cross-sectional vs. cohort vs. case–control) was not performed, as only one cohort study [36] and one case–control study [24] met the criteria for pooling, precluding a meaningful comparison against the nine cross-sectional studies.

**Figure 4:**
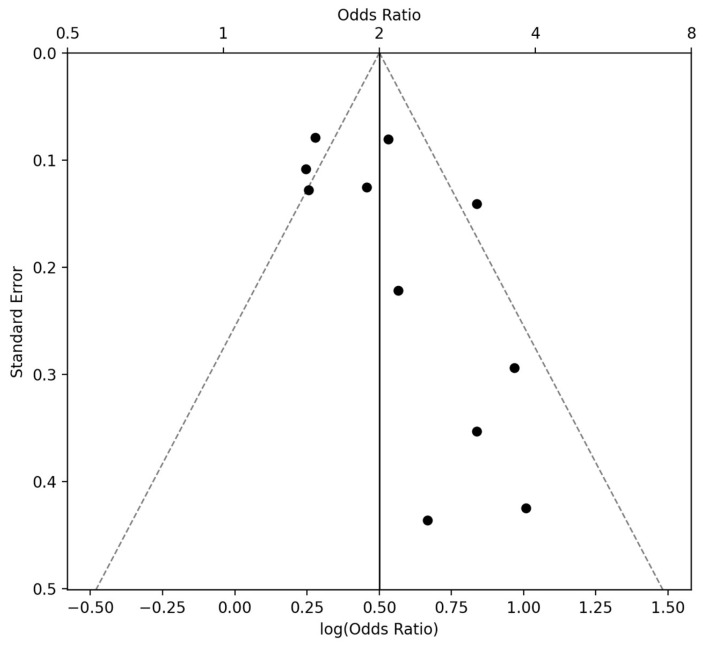
Funnel plot of the 11 studies included in the pooled meta-analysis, showing log(Odds Ratio) plotted against standard error, with pseudo-95% confidence limits centered on the pooled estimate (OR 1.65). Each dot represents an individual study; the solid vertical line indicates the pooled effect estimate; the dashed lines represent the pseudo-95% confidence limits.

Egger’s regression test showed no statistically significant evidence of funnel plot asymmetry (intercept = 1.73, t = 1.96, df = 9, *p* = 0.081), providing no strong indication of publication bias, although the modest number of pooled studies limits the power of this test to detect small-study effects (Figure 4).

### 3.6. Certainty of Evidence (GRADE)

The certainty of evidence for the pooled periodontitis–hypertension association was assessed using the GRADE framework and is summarized in Table 7. Starting from a baseline of Low, reflecting the exclusively observational nature of the included evidence, certainty was not further downgraded on any of the five assessed domains.

Risk of bias was not considered a serious concern, as none of the 11 studies contributing to the pooled estimate was rated high risk of bias on the design-specific Newcastle–Ottawa assessment (9 low risk, 2 moderate risk). Inconsistency was not considered a serious concern: although overall heterogeneity was substantial (I^2^ = 65%), a pre-specified subgroup analysis by periodontal case definition explained a considerable share of it (I^2^ = 48% among AAP/EFP-based studies and 0% among studies using alternative definitions), and the 95% prediction interval (1.10–2.48) did not cross the null. Indirectness and imprecision were not considered serious concerns, given a consistent exposure-outcome construct across studies and a comparatively narrow pooled confidence interval (1.42–1.93).

Publication bias was considered a serious concern and certainty was downgraded accordingly. Although Egger’s regression test did not reach statistical significance (*p* = 0.081), Begg’s rank-correlation test was significant (τ = 0.55, *p* = 0.020), and a clear small-study effect was identified: studies with a sample size below 3000 (*n* = 5) showed a pooled OR of 2.17 (95% CI: 1.77–2.67, I^2^ = 0%), compared with OR 1.47 (95% CI: 1.29–1.68, I^2^ = 51%) among larger studies (*n* = 6) (χ^2^ = 9.75, df = 1, *p* = 0.002). This pattern is consistent with either publication bias, selective reporting, or genuine effect-modification by sample size, and cannot be resolved with the present evidence base.

The overall certainty of evidence for the association between periodontitis and hypertension was therefore rated as Very Low. This rating reflects both the inherent limitations of exclusively observational evidence and a specific, identified concern regarding small-study effects, and should inform the strength of the interpretive language used when discussing the clinical implications of this association.

## 4. Discussion

This systematic review and meta-analysis examined the relationship between periodontitis and arterial hypertension across a broad range of epidemiological study designs, populations, and analytical approaches. The findings demonstrate a positive association between periodontitis and hypertension: individuals with periodontitis showed a 1.65-fold increased odds of hypertension (OR 1.65, 95% CI: 1.42–1.93) across the 11 studies reporting a directly comparable, adjusted estimate. Heterogeneity was substantial (I^2^ = 65%) but was partly explained by periodontal case definition, and the 95% prediction interval (1.10–2.48) did not cross the null. However, the overall certainty of this evidence, formally assessed using GRADE, was rated as Very Low, reflecting both the observational nature of the evidence and an identified small-study effect; this pooled estimate should therefore be interpreted as a signal warranting further investigation rather than a robust, settled finding.

The magnitude of the association observed in this meta-analysis is remarkably consistent with that reported in earlier systematic reviews. Martin-Cabezas et al. (2016) [10], pooling 16 studies, reported OR 1.50 (95% CI: 1.27–1.78), concluding that periodontitis was significantly associated with increased odds of hypertension, although with substantial heterogeneity and limitations inherent to observational designs. Similarly, Muñoz Aguilera et al. (2020) [9], pooling 40 studies, reported OR 1.22 (95% CI: 1.10–1.35) for moderate-severe periodontitis and OR 1.49 (95% CI: 1.09–2.05) for severe periodontitis, emphasized that the association persisted after adjustment for major confounders, supporting the hypothesis that periodontal inflammation may contribute independently to blood pressure dysregulation. The pooled estimate in the present review (OR 1.65, 95% CI: 1.42–1.93) falls within, and most closely matches, the severe periodontitis specific range reported by both prior reviews, which strengthens confidence in the approximate magnitude of the association across three independent reviews using different search periods and partially overlapping study samples.

This review adds to this prior evidence in four specific ways: four of the 11 pooled studies [32,37,53,54] were published after the search cutoffs of both prior reviews and were not available to them; a stricter effect-measure compatibility criterion was applied, explicitly excluding HR, RR, PR, IRR, and β-coefficient estimates from the OR-based pool rather than combining across measures; a formal, design-specific risk-of-bias assessment was applied across three separate NOS instruments; and a formal GRADE certainty-of-evidence rating is reported, which, to our knowledge, has not been presented by either prior review.

More recent narrative and epidemiological reviews have reinforced this association while highlighting persistent methodological challenges across the field, including inconsistent periodontal case definitions and residual confounding [56,57]. Separate systematic reviews focusing specifically on tooth loss as a proxy for periodontal disease [58,59] have also reported positive associations with hypertension, although effect sizes in that literature tend to be smaller and more heterogeneous, reflecting the multifactorial nature of tooth loss and its dependence on age, caries, socioeconomic factors, and access to dental care. Consistent with this distinction, tooth-loss-based studies identified in the present search were analyzed narratively rather than pooled with the clinically diagnosed periodontitis studies, to avoid conflating these related but non-equivalent exposure constructs.

While cross-sectional studies predominate in the pooled quantitative synthesis and cannot by design establish temporal precedence, complementary longitudinal evidence from studies employing Cox or Poisson regression models, offers a mixed picture of directionality. Two studies [42,47], in a large nationwide cohort and over five years of follow-up, reported a significant, positive association between periodontal measures and incident hypertension. In contrast, one study in the Health Professionals Follow-up [44], and in a nationwide cohort [52], found no significant association between periodontal disease and incident hypertension; another study [28] similarly found no significant association for periodontitis overall, with an elevated risk observed only within a subgroup with severe periodontitis. This mixed longitudinal evidence does not resolve the direction of the association, and the null findings from two of the largest available cohorts underscore the need for caution in interpreting the predominantly cross-sectional pooled estimate as reflecting a stable, unidirectional relationship.

The observed association between periodontitis and hypertension is biologically plausible and supported by a growing body of mechanistic evidence extending beyond observational epidemiology. Periodontitis is a chronic inflammatory condition characterized by a dysbiotic subgingival biofilm and a sustained host immune response, leading to elevated systemic inflammatory markers such as C-reactive protein, interleukin-6, and tumor necrosis factor-α [7,60]. Chronic low-grade inflammation plays a central role in the pathogenesis of hypertension by promoting endothelial dysfunction, arterial stiffness, oxidative stress, and altered nitric oxide bioavailability [61,62,63]. Supporting a potentially causal contribution of periodontal inflammation to vascular dysfunction, a randomized controlled trial demonstrated that non-surgical periodontal treatment improved endothelial function, measured by flow-mediated dilation, in patients with severe periodontitis [14]. More recently, a Mendelian randomization study using genetic variants as instrumental variables reported associations between antihypertensive drug-target genes and periodontitis, providing genetically informed evidence consistent with a mechanistic link between the two conditions that is less susceptible to confounding and reverse causation than conventional observational designs [11].

Several studies included in this review support these mechanistic pathways at the clinical level. Markers of active gingival inflammation, rather than attachment loss or probing depth alone, were independently associated with higher systolic blood pressure and hypertension prevalence, suggesting that current inflammatory burden may be more relevant to blood pressure regulation than cumulative historical tissue destruction [43,48]. Consistent with this, systemic inflammatory markers were reported to partially mediate the longitudinal association between periodontitis and incident hypertension, providing direct evidence for inflammation as a mechanistic pathway linking the two conditions [47].

Emerging evidence also implicates the oral microbiome and immune dysregulation in hypertension pathophysiology. Experimental and translational studies suggest that periodontal pathogens and their by-products may enter the systemic circulation via ulcerated pocket epithelium, contributing to vascular inflammation, endothelial activation, and dysregulated innate and adaptive immune responses [15,60]. More recent work describing oral–gut microbial transmission and immune-mediated vascular dysfunction further supports the concept that periodontal infection may exert systemic effects beyond the oral cavity [64]. While these mechanistic pathways cannot be directly tested within observational epidemiological designs, their biological coherence with the epidemiological findings reported here, together with the causal-inference evidence discussed above [11,14], supports a biologically plausible link between periodontitis and hypertension rather than a purely artefactual association.

Despite the consistent direction of association, substantial heterogeneity was observed among the pooled studies (I^2^ = 65%, Q = 25.59, df = 10, *p* = 0.004, τ^2^ = 0.04). This heterogeneity likely reflects differences across studies in periodontal case definitions, hypertension ascertainment methods, and population characteristics. A pre-specified subgroup analysis by periodontal case definition explained a substantial portion of the overall heterogeneity: studies applying the AAP/EFP classification (*n* = 5) showed a stronger, more homogeneous association (OR 1.90, 95% CI: 1.57–2.30, I^2^ = 48%) than studies using alternative periodontal indices (*n* = 6) (OR 1.35, 95% CI: 1.21–1.50, I^2^ = 0%), a statistically significant difference (χ^2^ = 9.42, df = 1, *p* = 0.002). This pattern suggests that stricter, standardized periodontal case definitions capture a more homogeneous form of periodontal disease that is more strongly associated with hypertension than broader or less standardized criteria.

Hypertension ascertainment method, geographic region, and risk-of-bias tier did not contribute significantly to the observed heterogeneity. Studies using an objective blood-pressure threshold, with or without an antihypertensive-medication criteria (*n* = 8), showed a pooled OR of 1.68 (95% CI: 1.37–2.06, I^2^ = 60%), similar to studies relying on self-reported diagnosis or treatment status alone (*n* = 3) (OR 1.65, 95% CI: 1.22–2.25, I^2^ = 82%; χ^2^ = 0.01, *p* = 0.94). Studies from Europe (*n* = 5, OR 1.64, I^2^ = 77%), Asia (*n* = 4, OR 1.76, I^2^ = 70%) and the two remaining studies from other continents (OR 1.61, 95% CI 1.27–2.03, I^2^ = 0%), showed similarly overlapping estimates (χ^2^ = 0.22, df = 2, *p* = 0.90). A sensitivity analysis restricted to the nine studies rated low risk of bias on the design-specific Newcastle–Ottawa assessment yielded a pooled OR of 1.60 (95% CI: 1.37–1.86, I^2^ = 66%), not significantly different from the two moderate-risk studies (χ^2^ = 2.50, *p* = 0.11), indicating that risk of bias did not materially drive the pooled result. By contrast, a subgroup analysis by sample size revealed a statistically significant small-study effect: studies with fewer than 3000 participants (*n* = 5) showed a markedly stronger, more homogeneous association (OR 2.17, 95% CI: 1.77–2.67, I^2^ = 0%) than studies with 3000 or more participants (*n* = 6) (OR 1.47, 95% CI: 1.29–1.68, I^2^ = 51%; χ^2^ = 9.75, df = 1, *p* = 0.002), a pattern discussed further in relation to publication bias in the Limitations below. Variability in covariate adjustment across studies may have further contributed to residual heterogeneity: most studies adjusted for age, sex, smoking, body mass index, and diabetes, but fewer accounted for physical activity, dietary factors, psychosocial stress, or systemic inflammatory biomarkers. A formal subgroup comparison between cross-sectional and cohort designs was not performed, as only one cohort study and one case–control study met criteria for pooling against nine cross-sectional studies, a distribution too imbalanced to support a meaningful design-based comparison.

From a clinical and public health perspective, these findings may have potential implications, although they should be interpreted with appropriate caution given the very low certainty of the underlying evidence. Hypertension is frequently undiagnosed or poorly controlled, and several studies included in this review reported a higher prevalence of undiagnosed or uncontrolled hypertension among individuals with periodontitis. This observation raises the hypothesis that dental professionals could contribute to the earlier identification of patients at increased cardiovascular risk, for instance by incorporating blood pressure screening into routine periodontal examinations, particularly in populations with limited access to healthcare. At present, however, this represents a potential opportunity worth exploring in future prospective and intervention studies, rather than a practice change supported by the current evidence base.

Conversely, these findings underscore the potential relevance of periodontal health within the broader context of cardiovascular risk, warranting further investigation. Although causality cannot be established from observational evidence alone, the single interventional study included in this review reported that non-surgical periodontal treatment reduced blood pressure in patients with refractory hypertension [51], and an independent randomized controlled trial demonstrated that periodontal treatment improved endothelial function, a key intermediate marker of cardiovascular risk [14]. These findings are hypothesis-generating rather than conclusive, given the very limited number and size of the available interventional studies, and any potential population-level cardiovascular benefit of periodontal treatment would require confirmation in larger, adequately powered randomized controlled trials before it could inform clinical or public health recommendations.

The strengths of this review include full adherence to PRISMA guidelines, a comprehensive multi-database search strategy, and a design-specific risk-of-bias assessment using three separate, validated Newcastle–Ottawa Scale instruments matched to study design (cohort, cross-sectional, case–control), rather than a single instrument applied uniformly across all designs. A further strength is the restriction of the primary quantitative synthesis to studies reporting a directly comparable, adjusted odds ratio for a single, explicitly defined exposure-outcome relationship, with studies using non-equivalent effect measures (hazard ratio, risk ratio, prevalence ratio, incidence rate ratio, or β-coefficient) or a reversed exposure-outcome direction analyzed narratively rather than statistically pooled. This review is also, to our knowledge, the first on this specific association to report a formal GRADE certainty-of-evidence assessment and a 95% prediction interval alongside the pooled estimate.

Several limitations must be acknowledged. Cross-sectional studies predominate among the 11 studies contributing to the pooled estimate (9 of 11), with only a single cohort study and a single case–control study meeting pooling criteria; this distribution precluded a formal subgroup comparison by study design and limits the ability to establish temporal precedence, so reverse causation cannot be excluded. A geographic overlap risk was identified between two pooled studies drawing on the same regional population [31,33], both from the Gävleborg County Public Dental Service, Sweden); a sensitivity analysis excluding each study individually confirmed that the pooled estimate was not materially affected (OR 1.71 excluding Holmlund et al.; OR 1.64 excluding Engström et al.), but patient-level data were not available to directly confirm or rule out participant overlap. Heterogeneity remained substantial even after subgroup analysis by periodontal case definition and hypertension ascertainment method, and the limited number of studies per subgroup (*n* = 3 to 8) precluded formal meta-regression. Finally, although Egger’s regression test did not reach statistical significance (*p* = 0.081), Begg’s rank-correlation test was significant (τ = 0.55, *p* = 0.020), and a statistically significant small-study effect was confirmed by subgroup analysis (χ^2^ = 9.75, df = 1, *p* = 0.002); this pattern contributed to a formal downgrade of the overall certainty of evidence to Very Low and indicates that the pooled estimate should be interpreted with considerable caution.

Future research should prioritize well-designed, adequately powered prospective cohort studies employing standardized, validated periodontal case definitions (e.g., the 2017/2018 World Workshop AAP/EFP classification) and objective hypertension ascertainment, to address the current imbalance toward cross-sectional designs and permit a meaningful comparison of cross-sectional and longitudinal evidence. Given the small-study effect identified in the present analysis, larger studies with transparent reporting, and ideally prospective trial or protocol registration, are needed to clarify whether this pattern reflects true effect-size heterogeneity, publication bias, or selective reporting. Individual participant data meta-analyses could also help resolve residual uncertainty arising from population overlap between studies conducted in the same geographic or administrative settings. Finally, adequately powered randomized controlled trials evaluating the effect of periodontal therapy on blood pressure outcomes, building on the preliminary evidence discussed above [14,51], are warranted to help clarify whether the observed association reflects a causal, modifiable relationship.

## 5. Conclusions

This systematic review and meta-analysis identified a consistent, statistically significant epidemiological association between periodontitis and arterial hypertension across 11 observational studies (OR 1.65, 95% CI: 1.42–1.93). Individuals with periodontitis showed increased odds of hypertension relative to those without periodontitis, consistent with a potential link between periodontal and cardiovascular health. However, the overall certainty of this evidence was rated Very Low using the GRADE framework, reflecting the exclusively observational nature of the included studies and an identified small-study effect, and this finding should therefore be interpreted as preliminary rather than conclusive.

Causality cannot be established from the observational evidence synthesized here, and substantial between-study heterogeneity, only partially explained by differences in periodontal case definitions and hypertension ascertainment methods, further limits the strength of any interpretation. Reverse causation and residual confounding cannot be excluded, particularly given the predominance of cross-sectional designs among the pooled studies. Nonetheless, these findings support periodontal status as a factor worth considering in the broader context of systemic and cardiovascular health, warranting further investigation.

From a clinical perspective, the potential relevance of periodontal health to cardiovascular risk merits further exploration in future research, and continued dialogue between dental and medical professionals is encouraged. However, the current evidence does not yet support the formal integration of periodontal assessment into cardiovascular risk evaluation protocols, and any such application should await confirmation from higher-certainty evidence.

Further well-designed prospective cohort studies and adequately powered randomized controlled trials are needed to clarify the temporal and potentially causal relationship between periodontitis and hypertension, and to determine whether periodontal treatment could contribute to blood pressure prevention or management.

## Figures and Tables

**Table 1:** PECOS, P: Patient; E: Exposure; C: Comparison; O: Outcomes; S: Study design.

PECO(S) Question for Inclusion Criteria		
P	(Patient)	Adults (≥18 years), with or without hypertension.
E	(Exposure)	Presence of periodontitis (diagnosed clinically by probing depth, clinical attachment loss, bleeding on probing, or validated periodontal case definitions).
C	(Comparison)	Individuals without periodontitis (periodontally healthy or without defined periodontal disease).
O	(Outcomes)	Arterial hypertension (defined by systolic/diastolic blood pressure thresholds, medical diagnosis, or antihypertensive medication use).
S	(Study design)	Observational studies, including cross-sectional, case–control, and cohort designs.

**Table 2:** Description of the included studies.

Authors (Publishing Year)	Study Design	Country	Sample Size (Female/Male)	Average Age in Years	Periodontitis Definition	Periodontal Examination	Definition of HTN	BP Assessment
Aguilera et al. (2021) [24]	Case–control	United Kingdom	500	35	AAP/EFP (2017)	Periodontogram	SBP ≥ 140 or DBP ≥ 90 mm Hg	Omron device M5-1 (3 measurements)
Ahn et al. (2015) [25]	Cross-sectional	South Korea	14,625 (8378/6247)	NS	CPITN	Periodontogram	SBP ≥ 140 or DBP ≥ 90 mm Hg or antihypertensive treatment	Mercury sphygmomanometer (2 measurements)
Al-Hasan et al. (2023) [26]	Cross-sectional	Egypt	376 (150/226)	NS	AAP/EFP (2017)	Periodontogram	SBP ≥ 140 or DBP ≥ 90 mm Hg or antihypertensive treatment	Automatic sphygmomanometer (3 measurements)
Ansari Moghadam et al. (2016) [27]	Cross-sectional	Iran	700	NS	≥10 lost tooth	Tooth loss	SBP > 140 or DBP > 90 mm Hg	Sphygmomanometer and Stethoscope (2 measurements)
Aremu et al. (2023) [28]	Prospective cohort	Puerto Rico/USA	540 (421/119)	49	CDC/AAP (2012)	Periodontogram	SBP > 140 or DBP > 90 mm Hg	Mercury sphygmomanometer and automated machines (2 measurements)
Chen et al. (2023) [23]	Cross-sectional	China	1488 (886/502)	64	AAP/EFP (2018)	Periodontogram	SBP > 140 or DBP > 90 mm Hg or self-reported diagnosis	Mercury sphygmomanometer (2 measurements)
Choi et al. (2015) [29]	Cross-sectional	South Korea	19,560	NS	CPITN	Periodontogram	SBP ≥ 140 or DBP ≥ 90 mm Hg or antihypertensive treatment	Mercury sphygmomanometer (2 measurements)
Darnaud et al. (2015) [30]	Cross-sectional	France	102,330 (48,095/54,235)	43.7	CPITN	Periodontogram	SBP ≥ 140 or DBP ≥ 90 mm Hg or antihypertensive treatment	Electronic device (TM-2541 A&D) (3 measurements)
Desvarieux et al. (2010) [22]	Cross-sectional	United States	657 (335/322)	55	CPITN	Periodontogram + salivary analysis	SBP ≥ 140 or DBP ≥ 90 mm Hg or antihypertensive treatment	Omron device M5-1 (2 measurements)
Engström et al. (2007) [31]	Cross-sectional	Sweden	1.239	53	CPI ≥ 4 = periodontitis	Periodontogram	SBP > 140 or DBP > 90 mm Hg	Electronic device (FUZZY-Logic) (2 measurements)
Gatarayiha et al. (2024) [32]	Cross-sectional	Rwanda	420	NS	CPI ≥ 4 = periodontitis	Periodontogram	SBP ≥ 140 or DBP ≥ 90 mm Hg or antihypertensive treatment	Omron device M5-1 (2 measurements)
Holmlund et al. (2006) [33]	Cross-sectional	Sweden	4254 (2388/1866)	53	SNDI	Periodontogram	Antihypertensive treatment	Self-report validated by medical records
Hosadurga et al. (2020) [34]	Cross-sectional	Malaysia	269 (150/119)	39	≥10 lost tooth	Tooth loss	SBP > 140 or DBP > 90 mm Hg	Mercury sphygmomanometer (2 measurements)
Iwashima et al. (2014) [35]	Cross-sectional	Japan	1643 (706/937)	66.6	CPITN	Periodontogram	SBP ≥ 140 or DBP ≥ 90 mm Hg or antihypertensive treatment	Standardized sphygmomanometer
Kawabata et al. (2016) [36]	Prospective cohort	Japan	2.588	NS	CPI ≥ 4 = periodontitis	Periodontogram	SBP > 140 or DBP > 90 mm Hg	Omron device M5-1 (2 measurements)
Könnecke et al. (2022) [37]	Cross-sectional	Germany	5934 (3026/2908)	59	Criteria of Eke and Page	Periodontogram	SBP ≥ 140 or DBP ≥ 90 mm Hg or antihypertensive treatment	Automated BP (2 measurements)
Machado et al. (2020) [38]	Cross-sectional	Portugal	1057 (610/447)	60.9	AAP/EFP (2018)	Periodontogram	SBP ≥ 140 or DBP ≥ 90 mm Hg or antihypertensive treatment	Automatic sphygmomanometer (1 measurement)
Mendes et al. (2021) [39]	Cross-sectional	Portugal	10,576 (6312/4264)	44.9	≥10 lost tooth	Periodontogram	SBP > 140 or DBP > 90 mm Hg	Automatic sphygmomanometer (1 measurement)
Ollikainen et al. (2014) [40]	Cross-sectional	Finland	1296	40	CPI	Periodontogram	SBP ≥ 140 or DBP ≥ 90 mm Hg or antihypertensive medication	Standard automated device (2 measurements)
Paddmanabhan et al. (2015) [41]	Cross-sectional	India	77	NS	CPITN	Periodontogram	SBP > 140 or DBP > 90 mm Hg	Mercury sphygmomanometer (2 measurements)
Park et al. (2024) [42]	Prospective cohort	South Korea	709,584	39.55	CPITN	Periodontogram	Antihypertensive treatment	Self-report validated by medical records
Peres et al. (2012) [20]	Cross-sectional	Brazil	1720 (955/765)	NS	≥10 lost tooth	Tooth loss	SBP > 140 or DBP > 90 mm Hg	Electronic sphygmomanometer (2 measurements)
Pietropaoli (2020) [43]	Cross-sectional	United States	8614 (4491/4123)	NS	AAP/EFP (2018)	Periodontogram	SBP ≥ 140 or DBP ≥ 90 mm Hg or antihypertensive medication	Omron digital device (3 measurements)
Rivas-Tumanyan et al. (2012) [44]	Prospective cohort	United States	31,543	76	CDC/AAP (2012)	Periodontogram	SBP ≥ 140 or DBP ≥ 90 mm Hg or antihypertensive medication	Mercury sphygmomanometer (3 measurements)
Rosa et al. (2023) [45]	Cross-sectional	Brazil	100	60.4	AAP/EFP (2018)	Periodontogram	SBP ≥ 140 or DBP ≥ 90 mm Hg or antihypertensive medication	Omron HEM-7120 (2 measurements)
Singh et al. (2016) [46]	Cross-sectional	India	1480	NS	≥10 lost tooth	Tooth loss	SBP > 140 or DBP > 90 mm Hg	Mercury sphygmomanometer (2 measurements)
Torrungruang et al. (2024) [47]	Prospective cohort	Thailand	901 (302/599)	58.5	CPITN	Periodontogram	SBP ≥ 140 or DBP ≥ 90 mm Hg or antihypertensive medication	Automatic device (2 measurements)
Tsakos et al. (2010) [48]	Cross-sectional	USA	13,994 (7377/6617)	NS	CPI ≥ 4 = periodontitis	Periodontogram	SBP ≥ 140 or DBP ≥ 90 mm Hg or antihypertensive medication	Mercury sphygmomanometer (3 measurements)
Umeizudike et al. (2022) [49]	Cross-sectional	Nigeria	150 (95/55)	50.5	CPI ≥ 4 = periodontitis	Periodontogram	SBP > 140 or DBP > 90 mm Hg	Omron digital device (3 measurements)
Uppal et al. (2024) [50]	Cross-sectional	India	80 (33/47)	53.8	Russell’s Periodontal Index	Periodontogram	SBP ≥ 140 or DBP ≥ 90 mm Hg or antihypertensive medication	Mercury sphygmomanometer and automated machines (3 measurements)
Vidal et al. (2013) [51]	Interventional	Brazil	26 (17/9)	53.6	CDC/AAP (2012)	Periodontogram	SBP ≥ 140 or DBP ≥ 90 mm Hg or antihypertensive medication	24 h ABPM (TM-2430)
Woo et al. (2021) [52]	Prospective cohort	USA	19,680 (8112/11,568)	51.8	NS	NS	SBP ≥ 140 or DBP ≥ 90 mm Hg	Self-report validated by medical records
Yang et al. (2025) [21]	Cross-sectional	China	144	NS	AAP/EFP (2017)	Periodontogram	SBP ≥ 140 or DBP ≥ 90 mm Hg or antihypertensive medication	Mercury sphygmomanometer (3 measurements)
Yildirim et al. (2022) [53]	Cross-sectional	Turkey	7008 (4211/2797)	31	AAP/EFP (2017)	Periodontogram	Antihypertensive treatment	Self-report validated by medical records
Zhan et al. (2023) [54]	Cross-sectional	China	13,195 (6620/6575)	56.4	AAP/EFP (2017)	Periodontogram	Antihypertensive treatment	Self-report validated by medical records
Zhao et al. (2019) [55]	Cross-sectional	China	3952 (1775/2197)	47	CPITN	Periodontogram	SBP ≥ 140 or DBP ≥ 90 mm Hg or antihypertensive medication	Mercury sphygmomanometer (2 measurements)

NS: Not specified; AAP/EFP (2017): American Academy of Periodontology and European Federation of Periodontology 2017 World Workshop; CPI/CPITN: Community Periodontal Index; SNDI: Swedish national dental insurance index; SBP: Systolic Blood Pressure; DBP: Diastolic Blood Pressure; ABPM: Ambulatory Blood Pressure Monitoring.

**Table 3:** Data extraction. OR: Odds ratio; RR: Risk ratio; PR: Prevalence ratio; HR: Hazard ratio; IRR: Incidence rate ratio; β: Beta coefficient; CI: Confidence interval; BP: Blood pressure; SBP: Systolic blood pressure; DBP: Diastolic blood pressure; HTN: Hypertension; AAP/EFP: American Academy of Periodontology/European Federation of Periodontology; CDC/AAP: Centers for Disease Control and Prevention/American Academy of Periodontology; CPITN: Community Periodontal Index of Treatment Needs; PPD: Probing pocket depth; BOP: Bleeding on probing; PISA: Periodontal inflamed surface area; ACC/AHA: American College of Cardiology/American Heart Association.

Authors (Publishing Year)	N	Effect Size (Type)	Estimate (95% CI or *p*-Value)	Adjustment/Covariates	Outcome	Association Direction	Reason if Not Pooled
Aguilera et al. (2021) [24]	500	OR	2.31 (1.17–4.67)	Severe periodontitis (AAP/EFP)	Undiagnosed hypertension (prevalent)	Periodontitis → HTN	—
Ahn et al. (2015) [25]	14,625	PR	1.10 (1.04–1.16)	Hypertension	Periodontitis (CPITN)	HTN → Periodontitis	Reversed direction
Al-Hasan et al. (2023) [26]	376	Mean difference	*p* = 0.005	Periodontitis (AAP/EFP)	Hypertension (prevalent)	Periodontitis → HTN	No multivariate adjustment
Ansari Moghadam et al. (2016) [27]	700	Mean difference	*p* < 0.001	Edentulism/tooth loss	SBP/DBP (continuous)	Tooth loss → BP	Exposure = tooth loss
Aremu et al. (2023) [28]	540	IRR	1.47 (1.01–2.17)	Severe periodontitis	Incident HTN/pre-HTN	Periodontitis → HTN	IRR not equivalent to OR
Chen et al. (2023) [23]	1488	OR	1.95 (1.13–3.37)	Periodontitis	Uncontrolled HTN (among hypertensives)	Periodontitis → BP control	Different outcome construct
Choi et al. (2015) [29]	19,560	β-coefficient	NR	Periodontal severity	SBP/DBP (continuous)	Periodontitis → continuous BP	β-coefficient, different outcome
Darnaud et al. (2015) [30]	102,330	OR	1.56 (1.35–1.80)	Composite oral-hygiene index	Hypertension (prevalent)	Not periodontitis	Not a periodontitis exposure
Desvarieux et al. (2010) [22]	657	β-coefficient	*p* < 0.05	Periodontal bacterial burden	SBP/DBP (continuous, over time)	Periodontitis → continuous BP	β-coefficient, different outcome
Engström et al. (2007) [31]	1239	OR	1.76 (1.14–2.72)	Severe periodontitis	Hypertension (prevalent)	Periodontitis → HTN	—
Gatarayiha et al. (2024) [32]	420	OR	1.95 (0.89–4.91)	Periodontitis (>5 mm)	Hypertension (prevalent)	Periodontitis → HTN	—
Holmlund et al. (2006) [33]	4254	OR	1.32 (1.13–1.54)	Periodontitis	Hypertension (prevalent)	Periodontitis → HTN	—
Hosadurga et al. (2020) [34]	269	β-coefficient	*p* = 0.250	≥10 missing teeth	Mean SBP	Tooth loss → BP	Exposure = tooth loss
Iwashima et al. (2014) [35]	1643	β-coefficient	*p* < 0.01	Severe periodontitis	Ambulatory BP (continuous)	Periodontitis → continuous BP	β-coefficient, different outcome
Kawabata et al. (2016) [36]	2588	OR	2.74 (1.19–6.29)	PPD ≥ 4 mm + BOP ≥ 30%	Hypertension (prevalent)	Periodontitis → HTN	—
Könnecke et al. (2022) [37]	5934	OR	1.28 (1.04–1.59)	Periodontitis (Eke/Page)	Hypertension (prevalent)	Periodontitis → HTN	—
Machado et al. (2020) [38]	1057	OR	2.31 (1.75–3.04)	Periodontitis (AAP/EFP)	Hypertension (prevalent)	Periodontitis → HTN	—
Mendes et al. (2021) [39]	10,576	OR	1.05 (1.03–1.06)	≥10 missing teeth	Hypertension (prevalent)	Tooth loss → HTN	Exposure = tooth loss
Ollikainen et al. (2014) [40]	1296	OR	0.98 (0.95–1.01)	Pockets ≥ 4 mm (continuous)	Hypertension (prevalent, ns)	Periodontitis → HTN	Continuous exposure
Paddmanabhan et al. (2015) [41]	77	Mean difference	*p* > 0.05	Periodontitis	Mean sitting SBP (continuous)	Periodontitis → continuous BP	No OR reported
Park et al. (2024) [42]	709,584	HR	1.10 (1.09–1.11)	Periodontitis (CPITN)	Incident HTN	Periodontitis → HTN	HR (Cox), not equivalent to OR
Peres et al. (2012) [20]	1720	β-coefficient	8.3 (0.1–16.7)	Total tooth loss	SBP (continuous)	Tooth loss → BP	Exposure = tooth loss
Pietropaoli (2020) [43]	8614	OR	1.58 (1.23–2.01)	Severe PISA (AAP/EFP)	High/uncontrolled BP (≥130/80, ACC/AHA 2017)	Periodontitis → HTN	—
Rivas-Tumanyan et al. (2012) [44]	31,543	RR	1.04 (0.98–1.10)	Periodontitis (CDC/AAP)	Incident HTN	Periodontitis → HTN	RR (Cox), not equivalent to OR
Rosa et al. (2023) [45]	100	OR	0.96 (0.93–1.01)	Hypertension	Periodontal/metabolic parameters	HTN → Periodontitis	Reversed direction
Singh et al. (2016) [46]	1480	OR	1.62 (1.12–2.35)	Self-reported tooth loss	Hypertension (prevalent)	Tooth loss → HTN	Exposure = tooth loss
Torrungruang et al. (2024) [47]	901	RR	1.17 (1.02–1.34)	Periodontitis	Incident HTN	Periodontitis → HTN	RR not equivalent to OR
Tsakos et al. (2010) [48]	13,994	β-coefficient	0.5 (0.3–0.6)	Gingival inflammation	SBP (continuous)	Periodontitis → continuous BP	β-coefficient, different outcome
Umeizudike et al. (2022) [49]	150	OR	3.31 (1.16–9.50)	Moderate gingivitis	Hypertension (prevalent)	Gingivitis → HTN	Not a periodontitis exposure
Uppal et al. (2024) [50]	80	β-coefficient	*p* = 0.001	Hypertension	Russell’s Periodontal Index	HTN → Periodontitis	Reversed direction
Vidal et al. (2013) [51]	26	Mean difference	*p* < 0.05	Periodontal treatment	BP change post-treatment	N/A (interventional)	Pre-post design, not observational
Woo et al. (2021) [52]	19,680	HR	2.26 (1.24–4.10)	Periodontitis	Incident HTN	Periodontitis → HTN	HR (Cox), not equivalent to OR
Yang et al. (2025) [21]	144	β-coefficient	10.08 (1.19–18.96)	Periodontal severity	Within-group dose–response	Within-group	No healthy control group
Yildirim et al. (2022) [53]	7008	OR	2.63 (1.48–4.68)	Periodontitis (AAP/EFP)	Hypertension (prevalent)	Periodontitis → HTN	—
Zhan et al. (2023) [54]	13,195	OR	1.70 (1.45–1.99)	Periodontitis (AAP/EFP)	Hypertension (prevalent, self-reported)	Periodontitis → HTN	—
Zhao et al. (2019) [55]	3952	OR	1.29 (1.00–1.65)	Periodontitis (CPITN)	Hypertension (prevalent)	Periodontitis → HTN	—

**Table 4:** Risk of bias assessment of cohort studies (Newcastle–Ottawa Scale). ★ indicates the criterion was met.

Study	Selection				Comparability		Outcome			Total (9/9)	Quality
	Representative of the Exposed Cohort	Selection of External Cohort	Ascertainment of Exposure	Outcome of Interest Not Present at the Start of the Study	Comparability of the Cohorts		Assessment of Outcomes	Sufficient Follow-up Time	Adequacy of Follow-up		
					Main Factor	Additional Factor					
Aremu et al. (2023) [28]	★	★	★	★	★	★	★	★		8	Low risk
Kawabata et al. (2016) [36]	★	★	★	★	★	★	★	★		8	Low risk
Park et al. (2024) [42]	★	★	★	★	★	★	★	★	★	9	Low risk
Rivas-Tumanyan et al. (2012) [44]	★	★	★	★	★	★	★	★	★	9	Low risk
Torrungruang et al. (2024) [47]	★	★	★	★	★	★	★	★	★	9	Low risk
Woo et al. (2021) [52]	★	★	★	★	★	★	★	★		8	Low risk

**Table 5:** Risk of bias assessment of cross-sectional studies (Newcastle–Ottawa Scale, adapted for cross-sectional design. ★★ = validated/objective clinical or laboratory measurement; ★ = self-report or non-validated method.

Study	Selection				Comparability		Outcome	Statistical Test	Total (10/10)	Quality
	Representative of the Sample	Sample Size	Non-Respondents	Ascertainment of the Exposure	Comparability of the Cohorts		Assessment of Outcomes			
					Main Factor	Additional Factor				
Ahn et al. (2015) [25]	★	★		★★	★	★	★★	★	9	Low risk
Al-Hasan et al. (2023) [26]		★		★★	★		★	★	6	Moderate risk
Ansari Moghadam et al. (2016) [27]	★	★		★	★		★	★	6	Moderate risk
Chen et al. (2023) [23]	★	★		★★	★	★	★★	★	9	Low risk
Choi et al. (2015) [29]	★	★		★★	★	★	★	★	8	Low risk
Darnaud et al. (2015) [30]	★	★		★	★	★	★★	★	8	Low risk
Desvarieux et al. (2010) [22]	★	★		★★	★	★	★★	★	9	Low risk
Engström et al. (2007) [31]	★	★		★★	★	★	★	★	8	Low risk
Gatarayiha et al. (2024) [32]	★			★★	★	★	★	★	7	Moderate risk
Holmlund et al. (2006) [33]	★	★		★★	★	★	★	★	8	Low risk
Hosadurga et al. (2020) [34]	★	★		★	★	★	★	★	7	Moderate risk
Iwashima et al. (2014) [35]	★	★		★★	★	★	★★	★	9	Low risk
Könnecke et al. (2022) [37]	★	★		★★	★	★	★	★	8	Low risk
Machado et al. (2020) [38]	★	★		★★	★	★	★	★	8	Low risk
Mendes et al. (2021) [39]	★	★		★	★	★	★	★	7	Moderate risk
Ollikainen et al. (2014) [40]	★	★	★	★★	★	★	★★	★	10	Low risk
Paddmanabhan et al. (2015) [41]				★	★	★	★	★	5	High risk
Peres et al. (2012) [20]	★	★		★	★	★	★	★	7	Moderate risk
Pietropaoli (2020) [43]	★	★	★	★★	★	★	★★	★	10	Low risk
Rosa et al. (2023) [45]				★★	★	★	★	★	6	Moderate risk
Singh et al. (2016) [46]	★	★		★	★	★	★	★	7	Moderate risk
Tsakos et al. (2010) [48]	★	★	★	★★	★	★	★★	★	10	Low risk
Umeizudike et al. (2022) [49]		★		★★	★	★	★	★	7	Moderate risk
Uppal et al. (2024) [50]				★★	★	★	★	★	6	Moderate risk
Yang et al. (2025) [21]	★	★		★★	★	★	★	★	8	Low risk
Yildirim et al. (2022) [53]		★		★★	★	★	★	★	7	Moderate risk
Zhan et al. (2023) [54]	★	★	★	★★	★	★		★	8	Low risk
Zhao et al. (2019) [55]	★	★		★★	★	★	★	★	8	Low risk

**Table 6:** Risk of bias assessment of case–control studies (Newcastle–Ottawa Scale). ★ indicates the criterion was met.

Study	Selection				Comparability		Exposure			Total (9/9)	Quality
	Case Definition Adequate	Representativeness of the Cases	Selection of Controls	Definition of Controls	Comparability of Cases and Controls		Assessment of Exposure	Same Method of Ascertainment for Cases and Controls	Non-Response Rate		
Aguilera et al. (2021) [24]	★	★	★	★	★	★	★	★		8	Low risk

**Table 7:** Summary of findings and certainty of evidence (GRADE) for the association between periodontitis and hypertension. ^1^ Overall I^2^ = 65%; largely explained by periodontal case definition (AAP/EFP subgroup I^2^ = 48% vs. other definitions I^2^ = 0%; test for subgroup differences *p* = 0.002); not explained by hypertension ascertainment, risk of bias, or geographic region (all *p* > 0.10); 95% prediction interval (1.10–2.48) does not cross the null. ^2^ Egger’s test not significant (*p* = 0.081), but Begg’s rank-correlation test significant (τ = 0.55, *p* = 0.020). A statistically significant small-study effect was confirmed by subgroup analysis: studies with N < 3000 (*n* = 5) showed OR 2.17 (1.77–2.67, I^2^ = 0%) versus OR 1.47 (1.29–1.68, I^2^ = 51%) for studies with N ≥ 3000 (*n* = 6), a significant difference (χ^2^ = 9.75, df = 1, *p* = 0.002). This pattern raises genuine concern for publication bias or selective reporting among smaller studies.

Outcome	N Participants	Study Design	Risk of Bias	Inconsistency	Indirectness	Imprecision	Publication Bias	Relative Effect (95% CI)	Certainty
Hypertension (periodontitis vs. no periodontitis)	48,761 (11 observational studies)	Cross-sectional (9), cohort (1), case–control (1)	Not serious	Not serious ^1^	Not serious	Not serious	Serious ^2^	OR 1.65 (1.42–1.93)	Very low

## Data Availability

The data presented in this study are available upon reasonable request from the corresponding author.

## Supplementary Materials

The following supporting information can be downloaded at: https://www.mdpi.com/article/10.3390/jcm15155930/s1, PRISMA 2020 Checklist [65].

## Abbreviations

The following abbreviations are used in this manuscript:

AAP/EFP	American Academy of Periodontology/European Federation of Periodontology
ABPM	Ambulatory Blood Pressure Monitoring
ACC/AHA	American College of Cardiology/American Heart Association
BMI	Body Mass Index
BOP	Bleeding on Probing
BP	Blood Pressure
CAL	Clinical Attachment Loss
CDC/AAP	Centers for Disease Control and Prevention/American Academy of Periodontology
CI	Confidence Interval
CPI/CPITN	Community Periodontal Index/Community Periodontal Index of Treatment Needs
CRP	C-reactive protein
DBP	Diastolic Blood Pressure
GRADE	Grading of Recommendations Assessment, Development and Evaluation
HTN	Hypertension
IL-6	Interleukin-6
IRR	Incidence Rate Ratio
NOS	Newcastle–Ottawa Scale
NR	Not Reported
OR	Odds Ratio
PD	Periodontal Disease
PISA	Periodontal Inflamed Surface Area
PR	Prevalence Ratio
PRISMA	Preferred Reporting Items for Systematic Reviews and Meta-Analyses
REML	Restricted Maximum Likelihood
RR	Risk Ratio
SBP	Systolic Blood Pressure
SNDI	Swedish National Dental Insurance Index
WBC	White Blood Cells
WHO	World Health Organization
